# Integrating mental health and non-communicable disease care: A WONCA advocacy project report

**DOI:** 10.4102/phcfm.v17i1.4875

**Published:** 2025-05-19

**Authors:** Stephen T. Engmann, Prince Ampofo, Christopher Dowrick

**Affiliations:** 1 Family Medicine Unit, Manna Mission Hospital, Accra, Ghana; 2 Community Health Department, Family Health Medical School, Family Health University College, Accra, Ghana; 3 Department of Psychology/Counselling Unit, Manna Mission Hospital, Accra, Ghana; 4 School of Psychology, Faculty of Health, Liverpool John Moores University, Liverpool, United Kingdom; 5 Department of Mental Health and Primary Care, Faculty of Health and Life Sciences, University of Liverpool, Liverpool, United Kingdom

**Keywords:** mental health, integrated care, non-communicable diseases, primary care, hypertension, type 2 diabetes mellitus, Ghana

## Abstract

The integration of mental health into the management of non-communicable diseases (NCDs) is crucial, particularly in low-resource settings like Ghana. This is a report of an integrated care project in primary care for the management of patients with hypertension and type 2 diabetes mellitus. This quality improvement project involved screening, providing information and education about common warning signs for mental health problems, and available health personnel from whom patients can seek help. This practice quality improvement project was executed in a primary care hospital in Ghana under the World Organization of Family Doctors (WONCA) Integrating Care Leadership and Advocacy Programme. Adult patients with hypertension and/or type 2 diabetes mellitus were screened using the 4-item Patient Health Questionnaire (PHQ-4) tool for anxiety and depression. The project screened 205 patients, of which 39 (19%) were found to have either anxiety and/or depression and were managed appropriately. The findings underscore the importance of integrating mental health care into the management of non-communicable diseases. Additionally, integration is essential to enhancing access to appropriate interventions and decreasing fragmentation in the delivery of care. This approach improves access to comprehensive care, reduces treatment fragmentation, lowers healthcare costs, fosters better patient satisfaction through holistic treatment, and reduces the stigma associated with mental health issues. This paper gives support to the feasibility of this integration in primary care settings. Several benefits have been demonstrated, showing the necessity of such integration in primary care settings, and advocating for policy with detailed guidelines for integrating mental health into non-communicable disease care in Ghana.

## Introduction

Non-communicable diseases (NCDs) and mental disorders are the leading causes of morbidity worldwide and account for three out of every 4 years lived with disability.^1^ Items 11–12 of the World Health Organization’s (WHO) 2013–2030 mental health action plan emphasise the complex relationship between mental disorders and NCDs with depression and schizophrenia increasing NCD-related premature deaths by 40% – 60%.^2^ Further evidence suggests that NCDs and mental disorders have similar predisposing psychosocial factors including low socioeconomic status, alcohol use and stress.^2^ Individuals living with NCDs in sub-Saharan Africa face challenges including a high prevalence of mental health issues such as depression and anxiety, scarcity of mental health professionals, a lack of integration of mental health treatments, stigma, limited access to services and financial barriers, highlighting the need for comprehensive and accessible care.^3^ How primary care provides mental health care and the effects this has on patients’ experiences with the disease are areas that require closer examination.^4^ The overall health and quality of life of NCD patients will improve when these chronic diseases are managed consistently.^5^ This is a report of a practice quality improvement project on the integration of mental health into care for NCDs in primary care.

## Project report

This practice quality improvement project was executed in an urban primary care facility in Ghana under the World Organization of Family Doctors (WONCA) Integrating Care Leadership & Advocacy Programme. The WONCA Integrating Care Leadership & Advocacy Programme was a training programme for young family physicians who want to support practice transformation by integrating behavioural health care into standard primary care practice.^6^ In collaboration with the Farley Health Policy Centre (FHPC) in Colorado and Columbia University, the programme was delivered by the WONCA Working Party on Mental Health and the WONCA Young Doctors Movement (YDM).^6^ The programme began on 02 August 2023 and lasted for 9 months. Participants of the programme were required to design and execute a project during the period of the programme. Participants were assigned mentors for their project to guarantee that every participant had access to a senior physician leader who takes part in the WONCA mental health workgroup and is dedicated to improving better treatment of patients with mental, emotional and behavioural issues.

The lead author and his primary care team executed this project at the Manna Mission Hospital, an urban primary care facility in Ghana. The hospital acts as a primary care facility in the community, by providing primary care services, and serving as a first point of care for patients. From the experience of the lead author in clinical practice, some of the patients with chronic diseases had mental health needs that were not well addressed, and thus the project sought to address these patient needs. By involving the target population through needs assessment, the project ensured that the care provided was comprehensive, culturally sensitive, and aligned with the patient’s needs and preferences, ultimately leading to better health outcomes and improved patient satisfaction. The project began in September 2023 and ended in April 2024. The project aimed to integrate mental health into the care of patients with NCDs in primary care.

The specific activities undertaken for the project were as follows:

- Hanging posters in patient waiting areas and consulting rooms of the facility, highlighting the common warning signs for mental health problems and where to seek help. Poster messages were made brief, easy to relate to and culturally relevant. The poster messages were further translated into Ga and Twi local languages.
- Adult patients 18 years and above with hypertension and/or type 2 diabetes mellitus were screened using the 4-item Patient Health Questionnaire (PHQ-4) for anxiety and depression.^7^ The screening was done from October 2023 to January 2024. Copies of the PHQ-4 tool were made available in all consulting rooms, and screening was incorporated into the consultation of patients with hypertension and/or type 2 diabetes mellitus. Screening was done by the doctors on duty at the outpatient department (OPD) and the specialised chronic care clinic. Patients who screened positive for anxiety or depression were further evaluated by the family physician or psychologist on the team for a definitive diagnosis, formulation and management.
- Health education through talks on mental health topics was held for patients in the waiting areas of the OPD, the Public Health Unit and the Specialised Chronic Care clinic of the facility in October 2023, January 2024 and April 2024.
- As part of the clinical meetings held in the hospital, two sessions on the biopsychosocial model of care and self-care of the health service worker were also held over the period of the project. A large part of such clinical meetings centred on discussions on how common mental health or psychosocial risk factors affect treatment adherence, lessons learned from multiple case studies and review of literature on integrated care. Based on these activities, the lead author piqued the interest of the primary care team on the need to address underlying comorbid mental health and psychosocial concerns in NCD patients.

The implementation strategies used to execute the project included engagement and collaboration with hospital management, healthcare providers and psychologist within the health facility, to ensure buy-in and support. There was also resource mobilisation, capacity building and training for doctors and healthcare staff. Integration was done into routine care for patients with hypertension and type 2 diabetes mellitus, making it a practical approach within the existing workflow. The project team tracked screening outcomes, referrals and patient responses to improve implementation as part of the monitoring and evaluation process. Clinical meetings provided a platform for discussion and continuous learning.

A needs assessment was conducted to evaluate the burden of mental health issues among patients with hypertension and type 2 diabetes mellitus at the facility. This included reviewing existing patient records and consulting healthcare providers on observed mental health concerns. Enablers, such as the presence of a dedicated family physician and psychologist, existing chronic care structures and hospital leadership support, were identified to facilitate smooth implementation. The progress of the implementation was assessed by tracking the number of patients diagnosed and receiving follow-up care. The cost implications were reviewed by considering expenses related to printing educational materials and screening tools. Midway into the project, a SWOT (Strength, Weakness, Opportunity and Threats) analysis was performed enabling informed decision-making on subsequent implementation.

## Project outcomes

Over the period of the project, a total of 205 patients with hypertension and/or type 2 diabetes mellitus were screened. The morbidity pattern showed that 51.7% had hypertension, 26.8% had type 2 diabetes mellitus and 21.5% had both type 2 diabetes mellitus and hypertension. Out of the 205 patients screened, 43 (21%) screened positive on the PHQ-4 with a total score of 6 or more and needed further evaluation. After further evaluation of the screen-positive patients, 39 (19%) were found to have either anxiety or depression and were managed by a collaborative team of professionals through patient-centred approaches. Out of this number, 8 had mild depression, 12 had moderate depression, 6 had severe depression and 3 had both generalised anxiety and depression. The remaining 10 had generalised anxiety disorder. Definitive diagnosis was followed by management which included pharmacological therapy and/or non-pharmacological therapy such as cognitive behavioural therapy based on the severity. Following management, 36 had resolution of symptoms and three transferred their care to other facilities because of relocation. Follow-up for all patients continued for up to 6 months from the time of the initial screening.

Among the strengths and enablers of the project was the use of a brief validated PHQ-4 screening tool which ensures accuracy and reliability in identifying mental health conditions. Appropriate information was easily accessible to individuals through mental health posters at vantage points. Among the weaknesses was the language barrier during consultations for some patients which made application of the tool challenging or required an interpreter. Additionally, screening during consultations may have increased workload for the doctors. The opportunities identified include the potential for scalability to other chronic diseases and training more healthcare providers in mental health care to strengthen integration efforts. The threats of the project were the negative perceptions of mental health issues which may limit patient participation and the presence of other pressing healthcare issues which may divert focus and resources from mental health integration.

## Discussion

### Benefits of integrating mental health into care for non-communicable diseases in primary care

This paper reported on a practice quality improvement project that was executed in an urban primary care facility in Ghana. It contributed to the expansion of care in patients with hypertension and type 2 diabetes mellitus by identifying and managing comorbid common mental health disorders including depression and anxiety in addition to routine medical care. Through an emphasis on the biopsychosocial model, primary care providers were also attentive to the psychosocial and/or mental health history of patients, supplementing the role of scarce mental health professionals in treating underlying common mental health conditions in patients with hypertension and type 2 diabetes mellitus.

Although the authors did not directly measure the cost-effectiveness of integrating care for mental health and the NCDs, studies have demonstrated that when primary care providers pay attention to and treat comorbid mental health conditions in hypertension and diabetes populations, it can potentially reduce the overall financial stress for the patient.^8,9^ The focus on the primary care facility (which is usually the first point of contact for patients with NCDs) helped to increase access to comprehensive care that addressed both medical and less severe or common mental health problems of patients. Severe mental health disorders including bipolar and schizophrenia were excluded from the project’s scope because they largely require specialised institutional care. Primary care providers are therefore well-positioned to make a difference for patients with NCDs suffering from common mood disorders that can often be overlooked.

### Lessons learned for policy and practice

- Continuous training and support for primary care providers are necessary to equip them with the skills to recognise and manage mental health conditions alongside NCDs.
- Successful integration in primary care requires collaboration among various stakeholders, including primary care physicians, mental health professionals and policymakers.

## Conclusion

Integrated care models in the management of NCDs and mental health comorbidities seem like a feasible approach with beneficial effects. This project report has given support to the feasibility of this integration in primary care settings. Truly integrating mental health and the management of NCDs in front-line primary care delivery will require much work from all stakeholders. To address the gaps at the policy level, there is a need for the Ministry of Health to develop detailed guidelines for integrating mental health into NCD care at all levels of the healthcare system.

The authors would like to thank the World Organization of Family Doctors (WONCA) for the opportunity to be part of the Integrating Care Leadership & Advocacy Programme, the WONCA Working Party on Mental Health and the WONCA Young Doctors Movement (YDM).
